# Use of urodynamics prior to surgery for urinary incontinence: How helpful is preoperative testing?

**DOI:** 10.4103/0970-1591.32065

**Published:** 2007

**Authors:** Gary E. Lemack

**Affiliations:** University of Texas Southwestern Medical Center, Dallas, TX, USA

**Keywords:** Urodynamics, stress urinary incontinence, abdominal leak point pressure, urethral pressure profilometry, maximum urethral closure pressure

## Abstract

It has not yet been definitively demonstrated that preoperative evaluation of women with stress urinary incontinence with urodynamic testing enhances presurgical counseling, more effectively models patients' expectations or improves postoperative outcome. Nonetheless, urodynamic testing is frequently utilized in the assessment of women with stress urinary incontinence and clearly accomplishes a number of goals when utilized for this purpose. For example, there are data to suggest that the risk of voiding dysfunction can be mitigated by utilizing data obtained from urodynamic testing to identify women more likely to void ineffectively after conventional stress incontinence procedures. Furthermore, it has been suggested though not proven, that patients with more severe forms of stress incontinence as identified by urodynamic testing, might be less likely to improve after surgery compared to others with more modest degrees of incontinence. Since urodynamic testing is invasive, costly and not always available, it is imperative that the usefulness of such testing be carefully explored and its utility appropriately defined. In this review, we discuss urodynamic techniques to assess stress urinary incontinence, particularly focusing on the ability of leak point pressure testing and urethral pressure profilometry to predict which patients would most likely benefit from surgery and which might be more likely to experience adverse events following surgery.

While the ideal procedure to treat stress urinary incontinence (SUI) may not yet be realized, surgical approaches have become more uniform and, in general, arguably more effective over the last decade. Efficacy rates for pubovaginal sling procedures (PVS) using rectus fascia and Burch colposuspension approach 90%, with success clearly varying depending on the definition of ‘cured’.[1] However, the morbidity associated with both these procedures, while in general not excessive, has precluded many from considering surgical intervention. Similar long-term efficacy rates have been noted for minimally invasive sling procedures, such as the transvaginal tape (TVT) and more recently, transobturator tape (TOT) procedures.[2,3] A standardized preoperative evaluation for women with SUI has not been universally adopted, but, at a minimum, most specialists mandate that the presence of SUI be visualized prior to surgery, with more formal evaluation with radiological testing and urodynamics utilized at the discretion of the surgeon.[4]

The extent to which a complete urodynamic (UD) evaluation impacts on the success of the treatment of SUI is a controversial issue. McGuire, in the initial description of abdominal leak point pressure measurement suggested that the severity of SUI, as measured by leak point pressure, might provide insight into the mechanism of SUI and direct therapy,[5] though both his group and others have more recently suggested that the sling procedure may be optimal for any type/severity of SUI.[6,7] Others have speculated that the finding of detrusor overactivity in addition to SUI during UD might alter the outcome following surgery, though a review of several studies on this topic did not reveal a clear consensus.[8] A more recent provocative study of the cost-effectiveness of preoperative testing for SUI revealed that UD offered little, if any, cost advantage over a noninvasive office assessment, particularly if the prevalence of SUI in the suspected patient population exceeded 80%.[9] Given the controversy surrounding the role of UD, it is not surprising that the use of UD is quite inconsistent. This finding was highlighted by a recent survey of physicians treating SUI, which revealed that a significant proportion did not use UD prior to surgery even if UD facilities were readily available.[10] Even patients with complaints of voiding dysfunction were not routinely tested prior to surgery.

When discussing urodynamic (UD) evaluation prior to incontinence surgery, there are several factors to consider that might potentially alter treatment planning or surgical decision-making. Among these are the ability of UD findings to predict SUI severity (for some physicians this may influence treatment recommendations), the relationship between preoperative SUI severity and treatment outcome (how successful can any surgery be given a particular degree of SUI severity) and the impact of other UD findings (detrusor overactivity, voiding dysfunction) on postoperative outcome.

## CAN SUI SEVERITY BE PREDICTED BY UD FINDINGS?

The two most commonly utilized measures to assess SUI in women are valsalva (or abdominal) leak point pressure (VLPP or ALPP) and urethral pressure profilometry (UPP). Difficulties in interpreting UPP data (the most common parameter assessed being maximum urethral closure pressure - MUCP) have been well described[11] and among many urodynamicists, UPP is difficult to consistently measure in reliable fashion. Abdominal leak point pressure may seem inherently more straightforward to measure and interpret, though there is certainly disagreement as to the best means of performing and reading leak point pressures.[12] For example, it is easier to obtain readings with valsalva maneuver than with cough and yet physiologically, more women are likely to leak with cough during UD testing.[13] Therefore, in general, there will be a significant number of women who symptomatically report SUI, but in whom, reliable VLPP data is not obtainable.[14] In general, if one is not able to obtain the data sought in a significant fraction of the patients complaining of SUI, the usefulness of the test must indeed be questioned. But apart from technical issues, there remains controversy as to whether either UPP or VLPP correlate with SUI symptom severity.

In patients with pure stress incontinence, the presence and severity of SUI can, in general, be reliably predicted by careful history-taking utilizing incontinence-specific questionnaires.[15] However, there is little evidence to support the contention that symptom severity correlates with either ALPP findings[16] or UPP,[17] though method of symptom assessment may influence these findings.[18] In other words, it is not clear whether patient perception of severity translates into physiologically more severe SUI based on ALPP or MUCP findings. Additionally, it has been demonstrated that patients without known risk factors for intrinsic sphincteric deficiency (ISD) have a surprising incidence of low ALPP, implying that assessing ALPP alone may not adequately discriminate those patients potentially at greater risk for failing conventional retropubic surgery, such as those patients with ISD.[19]

While few would argue that multichannel urodynamic testing provides the most comprehensive analysis of bladder function, it has been suggested that less invasive means may be able to discern the most severe forms of SUI. In fact, a positive supine stress test has been found to be highly predictive (97% PPV) of intrinsic urethral dysfunction,[20] though correlation with urethral closure pressure has been inconsistent.[21,22] Overall, in the setting of a negative supine stress test with a comfortably full bladder, severe ISD appears to be extremely unlikely. Therefore, if treatment planning is simply based on the presence or absence of ISD, it is possible that neither leak point pressure testing nor UPP may be necessary.

## CAN URODYNAMICS PREDICT PERSISTENCE OF SUI FOLLOWING SURGERY?

Lower preoperative MUCP has been associated with higher failure rates, particularly with retropubic procedures such as Burch colposuspension[23] or vaginal suspensions.[24] A randomized study comparing Burch and Marshall-Marchetti-Krantz (MMK) suggested that women with urethral hypermobility and low MUCP (< 20 cm H_2_O) were more likely to be cured by MMK, though the low patient numbers and short duration of the study make any definitive conclusion somewhat dubious.[25] Similar results were noted by a group studying predictors of success for a vaginal wall sling procedure.[26] Another recent study, by a group previously reporting inferior outcome with a Burch procedure for patients with low MUCP,[27] noted no impact of MUCP and equivalent outcome compared to pubovaginal sling using a modified Burch.[28] Furthermore, there does not appear to be solid evidence that patients with low MUCP fare worse after transvaginal tape procedures (TVT), as patients with recurrent SUI treated by TVT seem to do worse in the presence of a scarred and fixed urethra, independent of UPP findings.[29]

Given the different trajectory of the sling in transobturator tape procedures, some authors have suggested that urethral sphincter integrity may be important for this procedure to be successful. One study indeed showed that patients with MUCP above 30 cm H_2_ O fared better (continence rate of 86%) than those whose MUCP was lower (76%).[30] Others however, have shown equivalent early results, regardless of MUCP findings.[31] Taken together, these findings suggest that UPP is unable to discriminate who will fail most conventional open and transvaginal retropubic procedures and that more information is needed to determine if the success of transobturator procedures can be predicted by UPP testing.

While conflicting data exist, there appears to be no solid and consistent evidence demonstrating the ability of ALPP to predict the persistence of SUI following classic vaginal suspensions. So, while Kilicarslan and colleagues noted the combination of valsalva leak point pressure (VLPP) > 50 cm H_2_O and MUCP > 30 cm H_2_O to be associated with a success of 90% for *in situ* anterior vaginal wall sling, with a success of 65% for patients with lower VLPP and MUCP,[32] others have noted no significant impact of ALPP on treatment outcome following similar procedures.[33,34]

Similarly, in most studies, there is no apparent impact of leak point pressure findings on success following either a conventional fascial pubovaginal sling procedure placed at the proximal urethra/bladder neck or on distally placed synthetic slings, though conflicting data certainly exists. For example, Blaivas' group did not find that incontinence type (hypermobility versus ISD) to impact on surgical success, implying that VLPP findings were unlikely to be of prognostic significance.[6] Similarly, Morgan *et al* noted similar success rates among women with both Type II (91%) and Type III (84%) incontinence, again suggesting that the etiology and perhaps by inference, the severity of incontinence did not impact on efficacy.[35] More recently, Rodriguez *et al* studied the impact of UD findings on success following a distal polypropylene sling procedure for stress urinary incontinence.[36] While patients with lower VLPP used more pads and had more severe incontinence preoperatively based on questionnaire analysis (these findings have not been supported universally for women with SUI), they fared no worse following surgery than those with higher ALPP or those who did not leak at all on UD testing. Overall, success rates ranged from 92-95%, with little difference noted based on VLPP.

Other studies, however, have demonstrated that patients with very low VLPP (less than 60 cm H_2_O) were less likely to be cured following a TVT (0.6 risk) than those with higher LPP findings.[37] Since other surgical options might be considered for patients with very low VLPP and/or damaged urethras due to previous surgery,[38] clarifying the role of LPP assessment in predicting the successful treatment of SUI, particularly among patients traditionally considered at risk for failure (multiple previous procedures) would be of significant value.

## DOES THE FINDING OF DETRUSOR OVERACTIVITY ALTER TREATMENT OUTCOME?

The finding of detrusor overactivity on UD testing may impact on treatment planning and preoperative counseling. For example, one study demonstrated that those patients with mixed incontinence (both SUI and DO on testing) fared more poorly than those with pure SUI (69% vs. 97%), prompting that group to recommend preoperative cystometry.[37] In that study, however, the type of incontinence could be determined based on a minimal office-based screen and therefore prognostic information could have been given based on this screening alone and it was not clear that UD studies would have further improved either outcome or impacted treatment planning. Others have noted somewhat lower cure rates for SUI following TVT among patients with mixed incontinence preoperatively (confirmed urodynamically), though this difference did not achieve statistical significance (75% vs. 61% cure rate).[39] A more recent study of women treated with TVT, all of whom had coexisting DO and SUI on preoperative testing, noted essentially equivalent objective and subjective cure rates for SUI as has traditionally been reported for TVT in patients with pure SUI, suggesting that the finding of DO alone does not negatively impact on treatment efficacy.[40] Interestingly, 47% of the women were found to have stable bladders six months following TVT and 63% resolved their overactive bladder symptoms.

Given these somewhat conflicting findings, the value of performing cystometry to determine the presence of DO prior to surgery for SUI remains undetermined. There seems to be enough evidence to suggest that most patients with mixed incontinence will benefit from surgery such as a TVT. However, perhaps there are subsets of patients with more severe forms of DO or more urge predominant symptoms that would be more likely to fail. These parameters have yet to be solidly defined.

## UTILITY OF UD STUDIES IN PREDICTING VOIDING DYSFUNCTION AFTER SURGERY FOR SUI

Arguably the most life-altering and serious adverse outcome arising as a result of anti-incontinence surgery is the development of voiding dysfunction and frank urinary retention. Though early intervention can most often avoid long-lasting sequelae, this still means additional surgery and, frequently, persistent urinary incontinence. The ability of UD studies to accurately predict which women were at greatest risk for voiding dysfunction postoperatively would be a useful preoperative screening tool.

Among women who underwent PVS using allograft fascia, it was noted that four of 21 women who voided with no/minimal detrusor contraction during UD studies developed urinary retention, whereas no patients who voided with a detrusor contraction had difficulties voiding postoperatively.[41] Others have concluded that patients most likely to report *de novo* urgency, a possible manifestation of excessive tension, following TVT, were those in whom Valsalva voiding or detrusor hypocontractility was present preoperatively - though no direct pressure/flow comparison was made.[42] Still others have noted that patients suffering from dysfunctional voiding preoperatively (in this case defined as maximum free flow less than 12 ml/sec and detrusor pressure at maximum flow of greater than or equal to 20 cm H_2_ O) were more likely to have a lower objective cure rate (pad test) and lower quality of life scores following TVT than those that voided with normal pressure flow characteristics.[43] Wang and colleagues noted that both abnormal preoperative uroflow pattern and peak urinary flow rate of less than 15 ml/second predicted voiding dysfunction following TVT, though both of these parameters could have been collected without invasive testing.[44] Taken together, these studies imply that assessing voiding function, in most instances by performing a pressure-flow study, prior to anti-incontinence surgery can provide prognostic information regarding the likelihood of developing voiding dysfunction postoperatively.

## IMPACT OF ELIMINATING URODYNAMIC STUDIES PRIOR TO SURGERY

Since symptoms are often an unreliable indicator of urodynamic findings,[45] some have argued that performing UD studies might improve outcome following surgery, both by confirming the diagnosis and by establishing the presence of other concomitant diagnoses (such as voiding abnormalities or detrusor dysfunction) which might affect treatment outcome.[46] Overall, there seems to be little evidence to suggest that preoperative UD studies improve treatment outcome, regardless of the surgical treatment chosen. A recent Cochrane review focused on this very topic and found only two studies that met their criteria for inclusion, suggesting that there is insufficient data to either support or refute the importance of UD studies in predicting treatment outcome.[47]

Evidence from retrospective nonrandomized studies of women undergoing retropubic bladder neck suspension, some of whom had UD while others did not, suggest that UD had no impact on treatment success, as defined by postoperative questionnaire, so long as all patients were noted to have urethral hypermobility preoperatively.[48] It was suggested that patients who might have ISD (history of prior retropubic surgery, positive empty supine stress test, age greater than 50) undergo formal testing to address this concern, since they were considered to be more likely to fail bladder neck suspension. Others have investigated the outcome of TVT without preoperative UD evaluation and found no difference, overall, in terms of efficacy (cure rate of 87.7%), compared to previous studies where UD was routinely performed.[49] Currently, there is no solid evidence that eliminating UDS prior to performing incontinence surgery negatively impacts on patient outcomes for most patients planning surgery, particularly when noninvasive studies are utilized (such as uroflow and postvoid residual assessment) to help identify patients with preexisting voiding dysfunction.

## OTHER URODYNAMIC PARAMETERS TO ASSESS URETHRAL COMPETENCE

Recently, other parameters, such as urethral retro-resistance pressure (URP) have been studied as other means of assessing urethral strength and by inference, urinary incontinence in women.[50,51] In general, these studies have shown women with urodynamic SUI to have lower URP values than women without leakage during conventional urodynamics. However, URP was unable to discriminate patients with mixed incontinence (DO) and competent sphincters on urodynamic testing from those with urodynamically proven SUI. Furthermore, there was very little difference in URP values based on urinary symptoms. These data suggest that the utility of URP testing prior to surgical intervention is limited at this time.

## CONCLUSIONS

Urodynamic testing remains the most comprehensive tool in the evaluation of bladder and urethral function. Critical problems remain, however, regarding the use of UD testing, particularly in the preoperative evaluation of stress urinary incontinence in women. Among the most important of the problems are issues with standardization of technique, universally, in carrying out UD studies. It is not at all clear whether a comparison of UD variables (including both ALPP and UPP) between centers at this point is meaningful. Furthermore, while conflicting data exist, there is no consensus that preoperative UD testing improves patient selection or postoperative outcome for most women undergoing the most commonly performed procedures for stress incontinence beyond that which can be achieved by noninvasive office-based testing. Further assessment of the utility of UD testing on the impact on patient counseling and the influence on patient expectations following surgery is necessary to clarify the role of UD testing in the preoperative setting.
